# Primate origins of discourse-managing gestures: the case of *hand fling*

**DOI:** 10.1515/lingvan-2022-0004

**Published:** 2023-01-03

**Authors:** Pritty Patel-Grosz, Matthew Henderson, Patrick Georg Grosz, Kirsty Graham, Catherine Hobaiter

**Affiliations:** Super Linguistics Research Group, University of Oslo, Oslo, Norway; Wild Minds Lab, School of Psychology and Neuroscience, University of St Andrews, St Andrews, Scotland

**Keywords:** away gestures, interactive gestures, primate gestures, semantics, super linguistics

## Abstract

The last decades have seen major advances in the study of gestures both in humans and non-human primates. In this paper, we seriously examine the idea that there may be gestural form types that are shared across great ape species, including humans, which may underlie gestural universals, both in form and meaning. We focus on one case study, the *hand fling* gesture common to chimpanzees and humans, and provide a semantic analysis of this gesture.

## 1 Introduction

One of the perennial questions in linguistic description and analysis is whether there are universals of an explanatory sort. This question has recently gained new prominence in research on human gestures, as discussed in Cooperrider et al. (2018) and Cooperrider (2019). Relatedly, research on gestures in non-human primates has focused on the question of whether there are gestural form types or even gestural meanings that are shared across great ape species (Graham et al. 2018; Kersken et al. 2019). Finally, when it comes to the issue of meaning more broadly, as studied in natural language semantics, the question of abstract semantic universals that may be shared by all humans has been raised repeatedly, as in May (1991: 353), Partee (1992: 124–125) and von Fintel and Matthewson (2008). We propose a combined approach, which investigates universals in human gestures, great ape gestures, and semantics.

We build on the recent work on great ape gestures in Byrne et al. (2017) and Byrne and Cochet (2017), among others (e.g. Hewes 1973), who use comparative evidence across ape species. For instance, non-human great apes (chimpanzees [*Pan troglodytes*], bonobos [*Pan paniscus*], gorillas [*Gorilla*], and orangutans [*Pongo*]) appear to share more gestural form types (e.g., *Arm Raise*, *Reach Palm*, etc.) than would be expected by chance, suggesting a shared origin of gesture in apes. Qualities of this inherited system may have facilitated the emergence of language. Moreover, Graham et al. (2018) show that chimpanzees and bonobos also share the meanings of their gestures at a level above chance, indicating that such meanings may either be innate as well, or may arise across species based on shared cognitive needs and capacities.1As we will see when looking at concrete examples, we cannot exclude that shared meanings arise from mimesis or iconicity (i.e. imitation- or resemblance-based meaning), where a gestural prompt for a given action (e.g., “Move away”) would resemble an action (e.g., pushing or shooing the perceiver) that the signaller would carry out in a context in which the perceiver reacted satisfactorily. The question of whether iconicity can be found in great apes is highly controversial (see Byrne et al. 2017; Genty and Zuberbühler 2015; Moore 2014; Pika and Mitani 2006; Tanner and Byrne 1996; Tramacere and Moore 2018), and this paper does not aim to contribute on this topic. Humans share common ancestors with non-human great apes, and are in fact a closer relative to chimpanzees and bonobos than the gorillas and other great apes with whom chimpanzees share a large proportion of their repertoire (Byrne et al. 2017). As a result, these findings give rise to the expectation that the form types and meanings of shared great ape gestures are also present (and potentially innate) in humans. Kersken et al. (2019) demonstrate that the gestural form types shared by non-human apes are also found in the spontaneous gestures of 1-to-2 year old human toddlers. These findings give rise to the following two questions: Are the shared gestural form types also found in adults? If so, what are their meanings, and do they overlap with the meanings found in non-human great apes? We experimentally investigated these questions in Henderson et al. (2022). Henderson et al. (2022) is an experimental study that tests the extent to which core aspects of ape gesture meanings are retained and recognised when they are reproduced in human interactions. It did so by allowing participants to watch videos of these gestures reproduced in a naturalistic human interaction, and then select from among four potential meanings. However, Henderson et al. (2022) does not explore the linguistic implications at length; the purpose of this article is to go beyond Henderson et al. (2022), taking matters one step further. We lay out a conceptual framework for linking shared ape gestures to discourse-managing meanings in humans, proposing a concrete formalization of how gestures follow a complete transition from the communication of non-human primates, via correspondingly concrete gestures of humans, to the abstract discourse-managing gestures of humans. To do so, we proceed as follows.

We focus on the *hand fling* form type; *hand fling* is shared between humans and all of the non-human great ape species (chimpanzees, bonobos, gorillas, and orangutans), and may thus be a candidate for a gestural universal. In terms of its meaning, humans attribute the same physical “Move away” interpretation to this *hand fling* gesture that is present in other great apes, but also a more abstract discourse-managing meaning along the lines of “Forget it”. We briefly summarize the findings of Henderson et al. (2022) in Section 2, and then analyse a naturalistic use of *hand fling* as a discourse-managing gesture in Section 3. We argue that both interpretations share an abstract core meaning (Section 4); this overlap may derive from a shared abstract semantic building block, which in turn is a candidate for an abstract semantic universal in humans (Section 5), since gestures predate language even within the human lineage (see, e.g., McNeill 2012).

This paper systematically builds on the extensive literature on how abstract gestures are based in gestures with concrete meanings (e.g., Abner et al. 2015; Bavelas et al. 1992; Bressem and Müller 2017; McNeill 1992; Müller 2004), and on the rapidly growing literature on great ape gestures (e.g., Byrne et al. 2017; Graham et al. 2018; Kersken et al. 2019). Owing to both research traditions, it aims to make a contribution to both of them and to the connection between them, by adding formal semantic methodology with a focus on the form-meaning pairings associated with the *hand fling* gesture. Formal semantic approaches to animal communication constitute a new field of research (see, e.g., Schlenker et al. 2016), and this paper aims to expand the scope of this field to the comparison of great ape gestures and human gestures.

## 2 From chimpanzees to humans

In their research on chimpanzee gestures, Hobaiter and Byrne (2011: 749) define *gesture* as “discrete, mechanically ineffective physical movements of the body observed during periods of intentional communication” (compare, e.g., Kendon 2004: 7, Abner et al. 2015: 437–439, among many others, for definitions of *gesture* in humans). There is a general question of how to determine the meaning of a given gesture, both in humans and in other species; a human who gestures will often be hard-pressed to describe the meaning of a gesture in words (see, for example, the abstract gestural meanings in Bressem and Müller 2017, which amount to different types of ‘negative assessment’). This issue is amplified in the study of non-human primates, since human researchers cannot access intuitions about gestural meanings – they can only observe in which contexts the gestures seem to be appropriate. In their study of wild chimpanzees, Hobaiter and Byrne (2014) catalogue 39 uses of a *hand fling* gesture observed in five individuals, for which they assign the primary meaning “Move away” (73% of observations) and a secondary meaning “Stop that” (27% of observations).2See https://bit.ly/3Vj07rk for details. These researchers defined meaning here using a gesture’s *apparently satisfactory outcome*, which is established as follows. In non-human apes, we observe that a gestural *signaller* (defined as the individual gesturing) produces, and may repeat, a gesture until the *recipient* (for whom the gesture is intended) responds in a way that appears to satisfy the signaller. The behaviour that made the signaller stop gesturing is classified as the gesture’s *apparently satisfactory outcome* (Cartmill and Byrne 2007; Genty et al. 2009).

The *hand(s) fling* form is defined as a “Rapid movement of hand(s) or arm(s) from the signaller towards the recipient” in Byrne et al. (2017: 759), while the “Move away” meaning is defined as a prompt for the recipient to move away from the signaller (Hobaiter and Byrne 2014: 1598). *Hand fling* is present in all non-human great ape species (chimpanzees, bonobos, gorillas, and orangutans).3See https://greatapedictionary.ac.uk/gesture-videos2/ for example videos. Building on Kersken et al.’s (2019) finding that humans and other great apes overlap extensively in their gestural repertoire, our first question is whether the same *hand fling* can also be used as a *human* gesture with comparable meaning. Since the “Move away” meaning is strongly associated with *hand fling* in chimpanzees, we ask if humans make the same connection, i.e. would a human onlooker also associate a *hand fling* gesture performed by another human with a “Move away” meaning. (While a “Stop that” meaning has also been attested for *hand fling*, the fact that “Move away” is attested in more than 70% of occurrences is taken to indicate that *hand fling* is a ‘tight’ gesture strongly associated with the “Move away” meaning, Hobaiter and Byrne 2014: 1597.) Notably, since the gestures of non-human great apes are much more constrained in creativity than the gestures of humans, the optimal test scenario is one in which a human signaller performs the chimpanzee gesture as if it were a human gesture.

**Figure 1:**
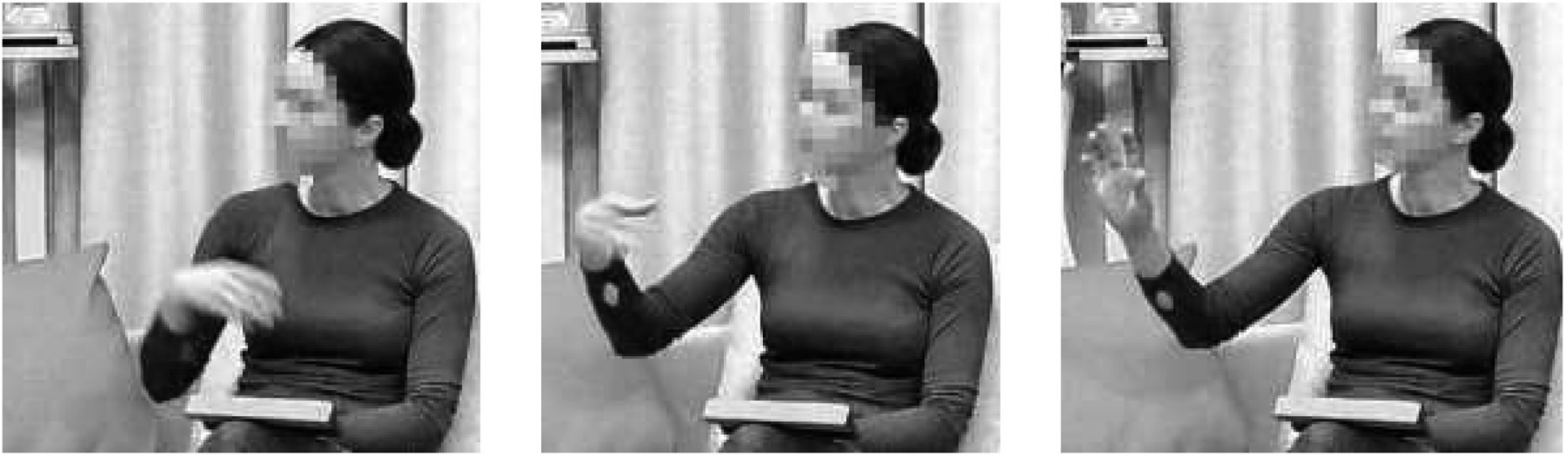
Three stills to illustrate the hand fling gesture.

Figure 1 is an illustration from a study presented in Henderson et al. (2022), that aimed to do this very thing; here, chimpanzee specialist Dr Catherine Hobaiter is depicted performing a *hand fling* gesture that adheres to its definition in chimpanzees but is performed as if it were a human gesture. Hobaiter aimed to reproduce ape-typical components of the gesture, e.g., the finger flexion and wrist position typical for chimpanzee hands, to ensure the gesture is faithful to what is found in chimpanzees, rather than anthropomorphised, to maximise similarity. Note that while human and chimpanzee vocal anatomy is markedly different (Nishimura et al. 2022), the physiology of human and chimpanzees in the limbs required for gesture production is not. So while the authentic reproduction of vocal signals would not be possible across species, it is possible to recreate ape gesture actions. By reproducing the chimpanzee *hand fling* gesture as if it were a human gesture, it is possible to test whether human onlookers draw the same meaning inference that is found in chimpanzees, namely a prompt for the recipient to “Move away”. This was indeed confirmed by our first experimental study (reported in Henderson et al. 2022), where 77% of 300 study participants chose “Move away” as the meaning of *hand fling* in a forced-choice task with four answer options. For the purposes of our study of *human* gestures that are isomorphic to chimpanzee gestures, this finding suggests that adult humans draw similar inferences from seeing such a gesture as is found in chimpanzees.

Naturally, the *source* of the “Move away” inference is unclear, i.e., whether the form-meaning association is innate, or simply acquired systematically due to shared cognitive mechanisms, which may also involve iconicity in the form of resemblance between the gesture and an actual activity of shooing away a person or animal. Either way, we expect the form-meaning association to be a candidate for a gestural universal – though possibly at an abstract level, which we discuss in Section 5. Moreover, the question of whether iconicity plays a role in the gestural form-meaning association is independent from Kersken et al.’s (2019) finding that the form of the *hand fling* gesture is found in 1-to-2 year old human infants; these infants will plausibly not have access to the same iconic resources as human adults.

In a next step, we observe the following: while gestures of the type in Figure 1 appear to be used exclusively to communicate about the external physical world by non-human great apes (i.e., “Remove yourself *physically*”), humans may assign additional meanings to such a gesture in addition to that physical meaning. We propose that an abstract discursive counterpart of “Remove yourself” may amount to “Remove the undesirable information that you have proffered”, or more casually, “Forget it”, or even “Nah”. In a second experimental study, we used the same forced-choice design with four answer options, but tested abstract discourse-managing meanings instead of the concrete chimpanzee meanings; we found that human participants can indeed infer a “Forget it” meaning from the same gesture in Figure 1. (“Forget it” was chosen by 95% of 300 study participants.)

While bearing in mind that Figure 1 is the controlled recreation of a chimpanzee gesture by a human signaller (rather than a spontaneous gesture performed as part of a realistic conversation), an inspection of the literature on gestures in humans reveals similarities to the ‘brushing away’ and ‘holding away’ gestures that Bressem and Müller (2014, 2017 discuss as part of their family of *Away gestures* (building on Kendon 2004: 248–264, Calbris 2011; see also Harrison 2010); they describe ‘brushing away’ as a gesture that resembles “Rapidly brushing away small, annoying objects” while ‘holding away’ resembles “Holding or pushing away an object” (Bressem and Müller 2017: 3). This is an important connection, as it substantiates the intuition that *hand fling* constitutes a universal gesture type in that such *Away gestures* have been found in a range of different languages and cultures. Note that in chimpanzees, the flinging motion of *hand fling*, an outward hand movement away from the body, is more important to the gesture’s definition than the final position of the hand; Bressem and Müller (2014: 1596) discuss such a movement, which they take to define their family of *Away gestures*, and connect it to “rejection, refusal, negative assessment, and negation”.

We will now outline the discourse-managing potential of *hand fling* in Section 3, and then return to a potential abstract core meaning in Section 4. At this point, it is worth noting that there seems to be cross-cultural variation in the perception of *hand fling*; native speakers of British, Canadian and American English report that *hand fling* as a discourse-managing gesture is quite rude, whereas native speakers of German find the gesture quite natural without a similar sense of rudeness; our discussion glosses over such differences.

## 3 Charting the discourse effects of *hand fling*

If we start by turning to the established formal semantics literature on discourse management, one concept that connects to the “Forget it” meaning of a *hand fling* gesture is the notion of *common ground management* in Krifka (2008: 246) (see also Repp 2013; Krifka 2017). The core idea is that we not only communicate to establish shared knowledge about a world/situation (see, e.g., Karttunen 1974; Lewis 1979; Stalnaker 1974), but we also communicate about how the discourse situation between us should evolve. To apply this notion to our *hand fling* gesture in its discourse-managing “Forget it” use, *hand fling* may communicate the signaller’s attitude on how a piece of information *φ* that has already been proffered by the recipient should be handled: *φ* should be rejected rather than being added to the common ground. This is in line with a treatment of *hand fling* as similar to Bressem and Müller’s (2014, 2017 ‘holding away’ gestures, which are characterized as “refusing and rejecting” information. Note that this discourse-managing information itself will, of course, be added to the common ground, e.g., in the shape of the proposition *the signaller wants the recipient to withdraw the information that the recipient had proffered*.

The *hand fling* with a “Forget it” meaning thus has a similar discursive effect to the expression of denial of a salient proposition (see, e.g., Repp 2013; van der Sandt 1991), thus preventing the targeted proposition *φ* from being added to the common ground. We can embed this in a discourse model as given in (1), using a notation from Repp (2013) for the sake of concreteness. (Compare Farkas and Bruce’s 2010 *Table* for a discourse model that would also permit a formal rendering of the contribution that *hand fling* makes to a discourse situation; see also Romero and Han 2004 for related research.) Example (1) incorporates the idea that a proposition (and thus a piece of information) *φ* is introduced as part of the statement in (1-i), the removal of which the signaller prompts in (1-ii). Compare Bressem et al.’s (2017: 176) description of the ‘holding away’ gestures as a means to “reject topics of talk, to stop arguments, beliefs, or ideas from intruding into the realm of shared conversation”.

(1)

**Table:** 

Discourse conditions for utterance u_n_ containing HAND-FLING:	
(i)	a preceding utterance u_n-1_ of the recipient_c_ has communicated that the proposition φ should be added to the common ground
(ii)	the signaller_c_ (who utters u_n_) prompts the recipient_c_ to remove φ from the discourse between the recipient_c_ and signaller_c_

We can now illustrate *hand fling* at work in a concrete example, (2)/(3) (inspired by a naturalistic example found in *The Fifth Child* [1988] by Doris Lessing [p. 40], and its official German translation by Eva Schönfeld). Here, *hand fling* occurs as a speech-accompanying gesture, rather than as a stand-alone gesture. We propose the following analysis: Robin’s utterance in (2a) triggers an inference (presumably at the level of conversational implicature) to *φ* = *we cannot manage*. Alex’s use of the *hand fling* gesture targets that inference (*hand fling* anaphorically takes *φ* as its propositional argument) and thereby rejects *φ*, in line with (1-ii). This strengthens Alex’s own assertion of *p* = *we* ***can*** *manage*.

(2)

**Table:** 

a.	Robin:	Our visitors are arriving tomorrow and the house is a mess!
b.	Alex:	We can manage ^HAND-FLING^

Such an analysis of (2) is corroborated by analogous facts from German. Consider the German translation in (3). In German, it is natural to add the discourse particle *schon* (see e.g. Egg 2012; Zimmermann 2018).

(3)

a.

**Table:** 

Robin:	Our visitors are arriving tomorrow and the house is a mess!

b.

**Table:** 

Alex:	*Das*	*schaffen*	*wir*	*schon* ^HAND-FLING^
	that	manage	we	PRT
	‘We will [*schon*] manage that.’

Egg (2012: 298) discusses an example similar to (3) (though not containing a gesture) and argues for an analysis that can be rendered as in (4): Robin’s utterance in (3a) communicates (simplified) *q* = *the house is a mess*, (4b). In (3b), Alex asserts the proposition *p* = *we will manage*, (4c), and adds the particle *schon*, which contributes the meaning inference in (4a), as applied to (4b–c) in (4d). (See Grosz 2021 for recent discussion of whether the non-at-issue contribution of German discourse particles, [4a]/[4d] is best modelled as a presupposition or designated use-conditional meaning.) Importantly, Alex’s retort to Robin’s statement serves to *cancel* a defeasible entailment (i.e. an entailment that arises in ‘normal’ circumstances), and *schon* (with the semantics in [4a]) highlights this cancellation, as spelled out in (4d). It is this defeasibly entailed proposition (*φ =* ¬*p*) that *hand fling* targets in (2b) and (3b).

(4)

a.

**Table:** 

⟦*schon*⟧(*p*)(*q*)	⤳ both *p* and *q* hold, and, according to the common ground, *q*
defeasibly entails	¬*p* (simplified from Egg 2012: 312)

b.

**Table:** 

*q = the house is a mess*	(asserted by Robin)

c.

**Table:** 

*p = we will manage*	(asserted by Alex)

d.

**Table:** 

⟦*schon*⟧(*p*)(*q*)	⤳ *the house is a mess (q)* defeasibly entails *we can not manage (*¬*p)*,
	but both *p (we will manage)* and *q (the house is a mess)* hold;
	therefore, the defeasible entailment (*q>*¬*p*) is cancelled.

It is worth noting that the *hand fling* gesture and the particle *schon* seem to reinforce one another in (3), as both of them serve to reject the salient inference *φ =* ¬*p = we cannot manage*. This behaviour is expected, given that both contributions may qualify as *use-conditional* in nature (Gutzmann 2013: 10–14), and it is a hallmark property of use-conditional meanings that they can be reinforced by repetition (cf. Potts’ 2007: 182–183 *repeatability* property of expressive content).4The combination of a *hand fling* gesture with the congruent discourse particle *schon* is not unlike the combination of several discourse particles in German (see e.g. Repp 2013: 248), as long as their meanings are compatible and potentially mutually reinforce each other. Having seen examples of the *hand fling* in its discourse-managing “Forget it” reading, (2b) and (3b), we now return to the question of how this may relate to the great ape meaning “Move away”, in Section 4.

## 4 Spelling out a semantic core of *hand fling*

The gestures of non-human great apes that have been described in the literature as of now (as in Hobaiter and Byrne 2014) are universally *directive* in the spirit of Searle (1975: 355), i.e., communicative acts by which the signaller prompts the recipient to perform some action or other. We can thus apply an established formalism for imperatives in human language to flesh out the meaning of *hand fling* in its “Move away” reading; for concreteness’ sake, we use Portner’s (2007: 358) formalism in (5). (See Kaufmann 2012, among many others, for alternative approaches to imperatives.) For a reader unfamiliar with formal semantic notation, (5) expresses that the meaning of *hand fling* in its “Move away” reading corresponds to the description of a property that holds of an individual *x* (by virtue of the lambda notation, *λx*). Specifically, this is a property of *x* removing physical closeness from *x* and the signaller in the context *c* in a situation *w* (as encoded in the text that follows the full stop in the second line of [5]). The part between colon and full stop (‘x = recipient_c_’) is a presupposition that is only met if the denoted property holds of the recipient in context *c*; this encodes the recipient-orientation of directives (such as imperatives). In Portner’s system, a directive of the type in (5) is communicated by a signaller with the intention to add the denoted property to the recipient’s *To-Do List*, a contextually given set of properties which the recipient is committed to realize of themselves. In the concrete case at hand, (5) amounts to a prompt for reducing physical closeness between the recipient and the signaller, thus increasing distance between them. (Bold type is added to facilitate comparison between [5] and [6].)

(5)

**Table:** 

⟦ *hand fling*_Move-away_ ⟧^c,g,w^
= [λx : x = recipient_c_ . x removes **physical closeness** from x and signaller_c_ in w]

At first encounter, (5) may seem like a round-about way of expressing “Move away”, but our aim is to see how this meaning could be mapped transparently onto a discourse-managing meaning such as “Forget it”. This mapping can be done by switching *physical closeness* with *information*, as shown in (6). The change from manipulating something physical in (5) to abstract information in (6) is reminiscent of the *conduit metaphor* that was applied to human gestures by McNeill (1992), based on Reddy (1979), which argues that the gestural manipulation of virtual objects can be transferred to an analogous manipulation of abstract information (see also Müller 2004; Abner et al. 2015: 439; Bressem and Müller 2017: 2–3 and Cooperrider et al. 2018: 14).

(6)

**Table:** 

⟦ *hand fling*_Forget-it_ ⟧^c,g,w^
= [λx : x = recipient_c_ . x removes **information** from x and signaller_c_ in w]

We can make the parallels between (5) and (6) even more explicit by abstracting over the theme argument and specifying its ontological type (*physical closeness* vs. *information*) in the denotation of the gesture. Such a restatement of (5) and (6) is given in (7) and (8), respectively; here, the gesture combines with a variable *i*, which amounts to a contextually salient object that either classifies as *physical closeness* (7), or as a piece of *information* (8). In the formal semantics, the value of *i* (i.e., the contextually salient object for which it stands in) is provided by a contextual assignment function *g*, in line with standard procedure.5A reader may wonder if it is possible to define a contextual variable *i* that can classify as *physical closeness* in (7), but *information* in (8), as these are ontologically quite different from one another; we maintain that similar issues arise with natural language expressions, e.g., see Asher’s (2011) discussion of the word *book*, which can denote an object (*a heavy book*) or a body of information (*an interesting book*).

(7)

**Table:** 

⟦ *hand fling*_Move-away_ *i* ⟧^c,g,w^
= [λx : x = recipient_c_ ∧ **physical-closeness**(g(i)) . x removes g(i) from x and signaller_c_ in w]

(8)

**Table:** 

⟦ *hand fling*_Forget-it_ *i* ⟧^c,g,w^
= [λx : x = recipient_c_ ∧ **information**(g(i)) . x removes g(i) from x and signaller_c_ in w]

With regards to the universals debate as outlined in Section 1, we can now reason as follows: First, we proposed that humans may share a set of universal gestures with chimpanzees (and other great apes); this has previously been shown for 1- to 2-year old human infants by Kersken et al. (2019); our experiments in Henderson et al. (2022) extend this inquiry to adult humans. Second, we have argued that abstract discourse-managing gesture meanings, in (6)/(8), are transparently derived from physical gesture meanings that communicate about the external world, given in (5)/(7), based on a shared semantic core meaning (here: *x removes g(i) from x and signaller*_*c*_). As a consequence, the mapping from shared great ape gestures to corresponding discourse-managing meanings should arise independently across different cultures, which would provide a new piece to solving the puzzle of where gestural universals come from (Cooperrider et al. 2018; Cooperrider 2019).

## 5 Broader consequences

On a big picture level, we propose that the meanings of shared great ape gestures may more broadly constitute primary building blocks of gestural meaning in humans. While these building blocks may be understood in a physical sense (“Remove yourself”, “Move away”), we proposed in Section 4 that they can ‘trickle down’ into the discourse-managing gestures of adult humans by virtue of a semi-predictable mapping (where “Remove yourself” becomes “Remove the undesirable information that you have proffered”, i.e., “Forget it”). If supported, this hypothesis would allow us to identify the origin of a subset of discourse-managing human gestures in shared great ape gestures. Returning to Hobaiter and Byrne (2014) with this goal in mind, Table 1 illustrates attested ape meanings (quoted from Hobaiter and Byrne’s 2014 supplemental materials).

**Table 1:** Excerpt of chimpanzee meanings from Hobaiter and Byrne (2014).

Meaning	Definition
“Acquire object”	recipient gives signaller object (e.g. food, leaf sponge, etc.)
“Attend to specific location”	recipient adjusts their behaviour to focus attention on the location indicated in the signaller’s gestural communication, usually in grooming
“Move away”	recipient moves away from signaller
“Move closer”	recipient moves closer to signaller
“Stop that”	the recipient either ceases behaviour previously directed towards the signaller or changes their behaviour to direct it towards another individual

If we take the meanings in Table 1 to form the basis of universal meanings in humans, we observe that some of them are negative/discouraging (such as the meaning “Move away”, which is associated with the *hand fling* gesture), while others are positive/encouraging (such as the meaning “Move closer”). Such notions of discouragement and encouragement may thus form the basis of universal gestural meanings. In the first instance, the universal meanings shared between humans and great apes will be physical; subsequently, discourse-managing uses will derive from some of these physical gesture uses (though not necessarily all of them).

**Figure 2:**
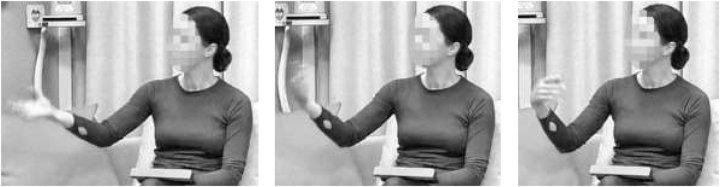
Three video stills to illustrate the beckon gesture.

To put this conjecture to the test, we can look at the *beckon* gesture (Hobaiter and Byrne 2014: 1598), which in chimpanzees is strongly associated with a “Move closer” meaning. In the same way in which “Move away” may map to *rejection of information*, we would expect that “Move closer” maps to *acceptance/solicitation of information*. In the second experimental study discussed in Section 2, we also presented human participants with a forced-choice task involving four answer options for possible meanings of the *beckon* gesture as performed in Figure 2. The majority of participants chose the answer option “Tell me more” (47%) or “Tell me about it” (35%),6The inclusion of two answer options with such a high degree of similarity was an artifact of the experimental design; there are good reasons to assume *post hoc* that participants may have converged on one of these options if only one of them had been included. with only 18% of participants choosing one of the two distractors, thus offering support for the suggestion that *beckon* may qualify as a positive counterpart of *hand fling* in discourse management as well.

## 6 Summary and conclusion

In this paper, we examined the idea that humans may share certain gestures with other great apes, such as chimpanzees, including the gestural form *hand fling* with an abstract gestural meaning that amounts to “Move away” (Section 2). As humans are not restricted to communicating about the physical external world, we proposed that the abstract gestural meaning of *hand fling* can be mapped onto the management of information in the discourse, by virtue of a simple change in the meaning of the gesture (Section 4). As proof of concept, we fleshed out a concrete application of this analysis to discourse situations in humans (Section 3). We concluded with a brief exploration of the broader consequences (Section 5). The central hypothesis generated in this paper amounts to the idea that parts of the meanings of shared great ape gestures (such as “Move away”) may be contained in those of the discourse-managing gestures of adult humans (such as “Forget it”); we consider this hypothesis to form the basis for future hypothesis testing in line with Henderson et al. (2022).
